# Editorial: The Role of RNA in Genome Stability: To Wreck or Repair?

**DOI:** 10.3389/fmolb.2022.848217

**Published:** 2022-02-09

**Authors:** Belén Gómez-González, Arijit Dutta, Wenyi Feng

**Affiliations:** ^1^ Departamento de Genética, Facultad de Biología, Universidad de Sevilla, Seville, Spain; ^2^ Centro Andaluz de Biología Molecular y Medicina Regenerativa-CABIMER, Universidad de Sevilla-CSIC, Seville, Spain; ^3^ Department of Biochemistry and Structural Biology, University of Texas Health Science Center at San Antonio, San Antonio, TX, United States; ^4^ Department of Biochemistry and Molecular Biology, SUNY Upstate Medical University, Syracuse, NY, United States

**Keywords:** DNA damage repair, genome stability, R-loop, DNA-RNA hybrid, transcription, DSB repair

It has long been known that the process of transcription is a source of genetic instability. RNA molecules have been implicated in both the generation of DNA damage and, in the recent years, in its repair. These seemingly conflicting roles of RNA motivated us to open this research topic covering transcription-associated genetic instability sources and the role of RNA in the repair of DNA damage.

The genome is challenged by both endogenous and exogenous sources of DNA damage. Additionally, failures in DNA metabolic processes, such as replication impairments or chromosomal miss-segregation, can threaten the transmission of the genetic information to the offspring by causing genome and chromosome instability. In particular, DNA transcription increases the frequency of mutation and recombination; this latter being caused, although not exclusively, by increased number of DNA double-strand breaks (DSBs), which are among the most harmful DNA lesions (Gaillard and Aguilera, 2016). RNA transcripts are constantly generated to supply for protein synthesis, RNA interference pathways, and regulation of transcription, translation, splicing as well as DNA repair processes via non-coding RNAs. Even heterochromatic regions can generate RNA molecules at telomeres (Azzalin et al., 2007; Luke et al., 2008; Schoeftner and Blasco, 2008) and centromeres (Saffery et al., 2003), that participate in the regulation of the telomeric (Fukagawa et al., 2004) and centromeric structures, thus impacting chromosome segregation and chromosome instability. The diverse roles of human centromeric RNA in chromosome stability were compiled by Leclerc and Kitagawa in this Frontiers Topic.

Nascent RNA molecules can potentially re-anneal with the DNA template to form DNA-RNA hybrids within ORFs (Huertas and Aguilera, 2003) and at transcription termination regions (Mischo et al., 2011) but also in telomeric (Luke et al., 2008) or centromeric regions (Kabeche et al., 2018; Mishra et al., 2021) and it is well established that DNA-RNA hybrids and R-loops, formed by the hybrid and the displaced single stranded DNA, are a major source of replication fork problems and ultimately DSBs (Gomez-Gonzalez and Aguilera, 2019). Thus, the odds of a transcribing DNA molecule to suffer DSBs are higher than a non-transcribing equivalent. In this Frontiers Topic, Long et al. describe how DSBs within a transcribed DNA causes a transient transcriptional shut-down but, at the same time, the DSB-induced transcripts are produced *de novo* and participate in DNA damage signaling (Francia et al., 2012; Michelini et al., 2017). The context in which the DNA breaks occur can affect the outcome of the repair mechanisms. Most of this effect is likely due to transcription-mediated changes in the chromatin status (Aymard et al., 2014; Clouaire and Legube, 2015). Notwithstanding, the potential impact of RNA molecules on DSB repair is emerging as a new controversial, but fascinating, field of study.

For instance, RNA molecules can impact DNA damage repair directly or indirectly by recruiting new protein factors involved in DNA damage signaling or repair. In fact, an increasing number of RNA-binding proteins appear to have a role in the DNA damage response, specifically to DSBs, as examined here by Klaric et al. Most of them were already studied for their functions in RNA metabolism, with the expected involvement in gene expression, but recently have been re-discovered to play additional functions in the regulation of DSB signaling and repair. It is of note that several RNA-binding proteins have an intrinsically disordered region (IDR), such as that in the Fragile X mental retardation protein (FMRP), which can function as an R-loop reader as shown by Dettori et al. in this Frontiers topic (Dettori et al.). Moreover, IDRs could facilitate dynamic assembly of protein complexes including R-loop resolvases such as DHX9 via liquid-liquid phase separation to promote R-loop resolution.

Adding a new layer of complexity, the affinity of RNA-binding proteins for RNA can be modulated by different posttranscriptional chemical modifications of the RNA molecule, creating a plethora of possible combinatorial epi-transcriptomic signatures that offer a high-level regulation to the DNA damage response. A prime example would be the N6-Methyladenosine (m6A), which, as reviewed by Qu et al. and Jimeno et al., modulates repair and genome stability (Qu et al.; Jimeno et al.). These review articles have summarized the recent findings on the role of RNA modifiers, such as RNA methyltransferase METTL3 and m6A RNA reader YTHDC1, in promoting DSB repair via homologous recombination (Zhang et al., 2020). Furthermore, the RNA molecule can also be edited, and A-to-I deamination via ADAR2 can also directly influence DSB repair by promoting R-loop resolution, facilitating DNA end resection to initiate homology-dependent repair (Jimeno et al., 2021a).

A growing number of reports have revealed that DSBs stimulate the formation of DNA-RNA hybrids (Aguilera and Gomez-Gonzalez, 2017) and some RNA-binding proteins can counteract or unwind the formation of hybrids at DSBs, as shown for Senataxin or DDX5 helicases among others (Cohen et al., 2018; Yu et al., 2020) (see Klaric et al. for a complete review). Strikingly, the DNA damage response can take advantage of this ability to promote efficient DNA damage repair, as exemplified by BRCA2 promoting DDX5 activity at damaged DNA (Sessa et al., 2021) or by BRCA1 interacting with Senataxin and the RNAi machinery to promote the repair of nicks at transcription termination pause sites (Hatchi et al., 2021). However, the role of DNA-RNA hybrids at DSBs remains controversial (Marnef and Legube, 2021). Whereas it has been put forth that accidental formation of the DNA-RNA hybrids at DSBs can interfere with homologous recombination repair (Ortega et al., 2021), it has also been suggested that hybrids are intermediates required for repair (Keskin et al., 2014; Ohle et al., 2016; D'Alessandro et al., 2018; Lu et al., 2018; Liu et al., 2021; Ouyang et al., 2021).

This Frontiers topic covers the most recent findings concerning the roles of RNA in genome stability and DNA repair mechanisms. We hope that the readers will find this collection of articles educational and useful for their own study to further advance this emerging field of research.
